# Using a flipped classroom teaching and learning approach to promote scientific literacy skill development and retention

**DOI:** 10.1002/2211-5463.13938

**Published:** 2024-12-03

**Authors:** Elaina B. K. Brendel, Ala Alzubi, Shrujan Rai, Christine Mariathasan, Laelie A. Snook, Jennifer M. Monk

**Affiliations:** ^1^ Department of Human Health and Nutritional Sciences University of Guelph Canada; ^2^ Kinesiology University of Guelph‐Humber Toronto Canada

**Keywords:** flipped classroom, learning approach, scientific literacy, skill retention

## Abstract

The development of scientific literacy (SL) skills is critical in the life sciences. A flipped classroom reverses traditional learning spaces such that foundational knowledge is acquired by students independently through recorded lectures and/or readings in advance of the lecture period and knowledge is consolidated through active learning activities in the classroom. A flipped classroom learning environment can promote critical skill development and knowledge application, and therefore, could enhance SL skill development. The objectives here were to (a) determine the effect of a flipped classroom learning environment on SL skill development in second‐year kinesiology students enrolled in a research methods course and (b) reassess SL skills 4 months later. SL skills were assessed using the validated test of scientific literacy skills (TOSLS) questionnaire at the start and end of the semester (*n* = 57) and reassessed 4 months later after the summer semester break (*n* = 46). During the flipped classroom semester, practical SL skills (TOSLS scores) were increased by 16.3% and TOSLS scores were positively correlated with the students' final grade (*r* = 0.526, *P* < 0.001). Four months later, average TOSLS scores significantly decreased compared to the levels at the end of the flipped classroom learning experience. Importantly, retention of SL skills (i.e., 4 months later TOSLS scores) were related to learning approach scores and were positively correlated with deep learning approach scores (*r* = 0.298, *P* = 0.044) and negatively correlated with surface learning approach scores (*r* = −0.314, *P* = 0.034). Therefore, SL skill retention was higher in students utilizing a deep learning approach (e.g., engaged, self‐regulation in learning, and seeking a deeper understanding of concepts) and lower in students utilizing a surface learning approach (e.g., limited engagement, rote memorization of concepts). Collectively, the results demonstrate the value of a flipped classroom in promoting SL skills while highlighting the role of students' learning approach in critical skill retention.

## Graphical abstract

Scientific literacy (SL) skill development and retention in kinesiology students was assessed in a research methods course taught using a flipped classroom approach. SL skills improved (16.3%, *n* = 57) with higher SL skill development positively correlated with final grade (*r* = 0.526). SL skills retention was positively correlated with a deep learning approach (*r* = 0.298) and negatively correlated with a surface learning approach (*r* = −0.314).

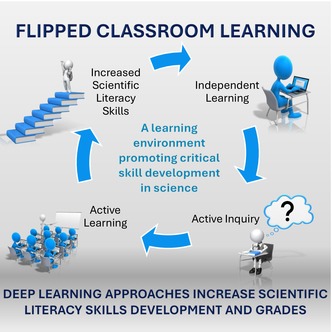

**Abbreviations**
SL: scientific literacy
TOSLS: test of scientific literacy

Scientific literacy (SL) encompasses a wide range of skills that reflect an individual's capacity to utilize and apply scientific knowledge to real‐world situations beyond the classroom. This range of SL skills includes, but is not limited to, acquiring and evaluating scientific information, critically analyzing and interpreting data, effectively communicating scientific findings, and applying scientific reasoning in problem‐solving [1, 2, 3]. To be scientifically literate, individuals must possess a rudimentary understanding of scientific vocabulary and constructs [4, 5]. This foundational knowledge can guide scientific inquiry through the careful evaluation of information, ultimately explaining scientific phenomena [5]. The majority of SL skill acquisition occurs within the classroom, with instructors playing a critical role in scientific knowledge transmittance [6]. At the undergraduate level, SL skills enable students to utilize their scientific knowledge and develop evidence‐based conclusions, engage in thought‐provoking conversations, and ask relevant questions within and beyond the classroom [2, 7]. In the broader context, SL skills are used to navigate complex science‐related issues and discern misinformation, which can help individuals make informed decisions when consuming media sources [2, 3]. Due to the wide range of components that constitute SL, a variety of approaches and pedagogies to assess SL skill competency have been used in undergraduate science education [1, 7].

Studies have shown that knowledge transfer and comprehension improve when students actively participate in the learning process [8, 9]. This is reflected well in a flipped classroom model, which incorporates self‐paced learning and enables students to identify their knowledge gaps that can be addressed during class time [10, 11]. The flipped classroom teaching approach can provide a foundation for knowledge acquisition and encourages students to utilize a wide variety of learning techniques [12, 13]. It prioritizes a student‐centered approach to learning through interactive engagement or active learning with peers and problem‐based learning [8, 12, 14, 15]. Traditional didactic lectures have been criticized for the passive transfer of knowledge, whereas the flipped classroom model has been shown to transform the classroom space into an environment that promotes students' reflection, critical thinking, and reinforcement of learning objectives [12, 14]. Direct instruction lecture components are moved online, and students are expected to independently familiarize themselves with course material through video‐recorded lectures and additional resources provided by the instructor prior to class time [15, 16, 17, 18, 19, 20, 21]. This creates flexibility and enables students to learn at their own pace and revisit complex or abstract concepts multiple times, leading to a deeper understanding and better retention of the material [1, 2, 3, 22, 23, 24, 25].

The pre‐lecture phase removes the time constraints that instructors typically encounter in a traditional lecture format, by allowing students to engage in higher levels of thinking, such as reasoning and problem‐solving, within the classroom environment [26]. Thus, the in‐class time of a flipped classroom instructional approach can focus on engaging learning methods, such as collaborative and interactive learning activities, group work, and discussions that help to reinforce concepts learned individually, leading to a deeper understanding of course concepts [23, 27] and promotes the development of important lifelong learning skills like critical thinking, creativity, communication, and problem‐solving [19, 22, 23, 28, 29, 30, 31, 32]. Additionally, this teaching and learning environment fosters interpersonal communication and can strengthen the student–instructor relationship [8, 12, 15, 17]. A recent meta‐analysis determined that students in flipped classroom settings often achieve higher examination scores and better overall course grades [33]. Although there is variability in the effect of a flipped classroom on academic performance, there are other benefits associated with this instructional model beyond grades [22, 28]. The flipped classroom has been shown to facilitate the development of higher‐order cognitive skills (application, analysis, synthesis) and enhance problem‐solving abilities through integrative tasks and active learning methods [24, 34]. Greater instructor feedback is also facilitated, as instructors have more opportunities to provide feedback during in‐class sessions, enhancing the learning experience [22, 23, 28]. Additionally, the flipped classroom model encourages students to take greater responsibility for their learning, develop self‐management skills, and enhance their ability to regulate their own learning processes [22, 24]. Consequently, students experience higher levels of satisfaction and engagement, attendance rates, and assignment submission rates [24, 28].

A flipped classroom requires a substantial investment of time and resources from instructors, students, and institutions [24]. Instructors need to be aware of the possible challenges encountered by students and provide support by adapting to their role as facilitators and mentors in the learning process, therefore, additional instructor training may be required [24]. Implementing a flipped classroom learning environment can present various challenges for students adapting to a learning environment that may be unfamiliar with. This teaching approach may require students to recall a larger amount of prior knowledge for effective learning and comprehension of complex concepts may be difficult for some students, therefore, additional resources may be required to ensure that the out‐of‐class learning is effective [24]. Student feedback about a flipped classroom learning environment is generally positive, however, the significant time investment required in courses using this type of learning environment has been shown to increase feelings of pressure, primarily amongst students enrolled in rigorous undergraduate programs [8, 12, 15, 17, 35]. There is also skepticism about the flipped classroom learning environment amongst students who are accustomed to traditional lecture‐based passive teaching methods centered around instructor dependence; thus, highlighting the need to develop strategies to successfully integrate students into the flipped classroom style of instruction [12, 26]. Ultimately, regular feedback through student surveys, direct communication with instructors, and continually augmenting course delivery can help students adapt to the flipped classroom learning environment [36]. Another challenge for students is the amount of time needed for viewing the pre‐class video lectures and the requirement for high levels of self‐regulation and motivation to learn [23, 24]. To overcome these obstacles, it is recommended to incorporate quizzes at the beginning of in‐person classes and/or to keep the length of videos limited (e.g., 60 min) and available to students 1 week before the accompanying in‐class learning activities [17, 23, 26].

The flipped classroom instructional model has been used in multiple disciplines and can potentially aid students in their transition from the classroom to the professional workforce [18, 33]. In this connection, the improved accessibility and dissemination of educational videos amongst different institutions may facilitate curricular standardization, as observed in several medical specialization rotations [15]. In kinesiology, which is a regulated health profession in Ontario, Canada, practitioners use SL skills in evidence‐based practice, however, literacy and numeracy skills were deficient in students evaluated from four Ontario universities [37]. This highlights the need for effective instructional approaches to promote SL skill development in students in undergraduate kinesiology programs, but more broadly across science majors. The objective of this study was to determine the effect of a flipped classroom learning environment on SL skill development in second‐year kinesiology students in a course dedicated to SL skill acquisition. A secondary objective was to assess SL skill retention 4 months after the flipped classroom learning environment.

## Methods

### Course delivery and flipped classroom teaching approach

The emphasis of the course Research Methods for Kinesiology (SCMA*2110) is on understanding different types of research designs and the scientific method. The course was scheduled in a 12‐week semester with a single weekly lecture that was 2 h and 45 min in duration. In contrast to previous academic cohorts, where the course format consisted of traditional lectures, the Winter 2023 learning environment was modified for the flipped classroom approach. Students were provided with a pre‐recorded lecture of approximately 45 min in duration, 1 week in advance of the in‐person scheduled lecture when the material would be discussed. The scheduled class time was reduced to 2 h in length to incentivize watching the 45‐min pre‐recorded lectures in advance of the scheduled class time. The in‐person class time was divided into three parts: (a) a brief review of the main topics covered of the lecture recording that was informed by a weekly ‘muddiest point’ learning reflective assignment described below (15–30 min), (b) active learning activities including simulations, case studies, group discussion questions in break out groups (60–75 min), and (c) full‐class discussion, questions, and reflection on the activities (15–30 min). Course assessments were worth 50% of students final grade and consisted of (a) weekly learning reflective assignments (5%), (b) CONSORT evaluation of primary research (2.5%), (c) completion of the research ethics online modules developed by the Tri‐Council Agency of Canada (required training for any research involving human participants in Canada, 2.5%), (d) experimental design evaluation assignment (group discussion and individual evaluation submission, 5%), (e) practical SL skill evaluation (completed at start and end of semester with the highest score earned worth 5%; required course component also used in the research project, described below), (f) kinesiology study design assignment (10%), (g) mini‐research proposal and participation consent form assignment (20%; 15% for the research proposal and accompanying consent form and 5% for the peer evaluation and critical feedback component).

The assignments were scaffolded throughout the semester to guide students from simple identification of the elements of a research study toward designing a cited research study proposal and creating an informed consent letter, wherein Bloom's Revised Taxonomy was used to guide the semester long progression of cognitive process complexity [38]. The weekly learning reflection was a brief ‘muddiest point’ assignment, in which students were required to identify the topic/idea that they found the most difficult in the lecture recording and to identify why it was difficult to understand. This was due before the start of the class in which the topic was discussed and was used by the instructor to inform which concepts would be reviewed in‐class with the entire class. Examinations constituted the other 50% of students grades in the form of (a) two noncumulative term tests (each worth 10%, written during week 5 and 9 of the semester) and (b) a cumulative final examination (30%). Both the term tests and final examination were a combination of multiple choice and short answer (50% of total marks for each type of question). Short answer questions included definitions, identifying components of a research study, designing a research study, and critically evaluating the validity and ethics of a published research study. The weighting of higher‐order cognitive process questions increased throughout the semester and was guided by Bloom's Revised Taxonomy [38].

### Participants and study design

**Fig. 1:**
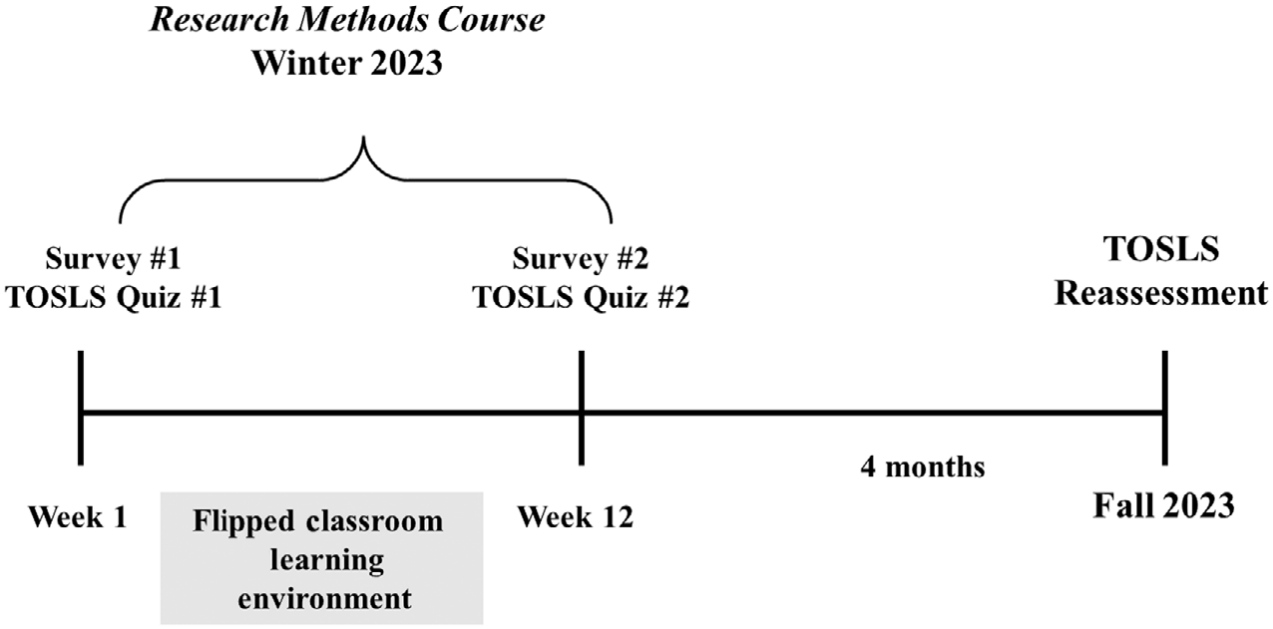
Study timeline depicting the flipped classroom learning environment in the Research Methods course in the Winter 2023. Practical scientific literacy skills were assessed using the Test of Scientific Literacy Skills (TOSLS) in an online quiz that was concurrent with the online surveys at the start and end of the semester (weeks 1 and 12, respectively). Practical SL skills were reassessed using TOSLS 4 months after completion of the course.

This project was approved by the University of Guelph Research Ethics Board (REB#22‐07‐001) and all participants, who were students in the Kinesiology Program at the University of Guelph‐Humber, provided their written informed consent. All students in the program take the same courses in each semester as they progress through the program. In the winter semester, students take the course Research Methods for Kinesiology (SCMA*2110), which was taught in a flipped classroom format. A summary of the overall study design is shown in Fig. 1. Students were invited to participate in the research project by completing two online surveys (described below), one at the start of the semester (during week 1) and one at the end of the semester (during week 12). Additionally, students in the course completed a SL practical skills assessment quiz at the start of the semester (week 1) and again at the end of the semester (week 12), which was a required component of the course (with the highest score earned worth 5% of student's final grade). Therefore, when students provided their informed consent to participate in the research project, they completed the online surveys and permitted the research team to access their grades in the course to be used for research purposes. Of the 85 students enrolled in the course, 57 provided their informed consent and completed both online surveys (Survey 1 and Survey 2) and both SL practical skill assessment quizzes (as the start and end of the semester). This is reflective of 67.1% participation. The students that participated in the research project had similar academic outcomes in the course compared to the entire class (includes both study participants and those who abstained from study participation). The average final grade in the course for all students enrolled in Research Methods was 76.8%, whereas the final grade in the course for the cohort of students that participated in the research project was 78.9% (unpaired *t*‐test, *P* = 0.16), indicating that there was no significant difference in final academic outcomes between the students that participated in the study and the overall class average.

### Online surveys

The online surveys were identical, administered at both the start and end of the semester, and assessed students' (a) perceived SL capabilities using assessment questions published previously [39, 40, 41], (b) learning approach (namely deep versus surface learning approaches) using the validated revised two‐factor study process questionnaire [42], and (c) researcher generated questions pertaining to students' perceptions of the significance of scientific literacy in kinesiology professional settings. The online surveys were administered using the Qualtrics Insight Platform (Provo, UT, USA) and invitations to participate were distributed using a private link to the student's institutional email address. Participation was optional, and a participation incentive was provided for completing Survey 1 (2% bonus added to students' midterm examination grade) and Survey 2 (2% bonus added to students' final examination grade). Alternative assignments were provided for students who did not want to participate in the online survey but still wanted to earn the participation incentives.

### Practical SL skill assessment

The practical SL skill assessment completed at the start and end of the semester (during weeks 1 and 12, respectively) and aligned with online survey data collection and consisted of the validated questionnaire called the Test of Scientific Literacy Skills (TOSLS) [1]. TOSLS is comprised of 28 multiple choice questions divided into nine SL skill categories including the ability to (a) identify a valid scientific argument, (b) evaluate the validity of sources, (c) evaluate the use and misuse of scientific information, (d) understand elements of research design and how they impact scientific findings/conclusions, (e) create graphical representations of data, (f) read and interpret graphical representations of data, (g) solve problems using quantitative skills, (h) understand and interpret basic statistics, and (i) justify inferences, predictions, and conclusions based on quantitative data. Students' TOSLS scores (at the start and end of the semester) were used in the research project (along with their responses to the online survey questions), and their highest TOSLS score earned during the semester was worth 5% of their final grade. This was intentional to reward skill growth and to avoid penalizing students that started the course with lower practical SL skills, which was particularly important given that SL skills are formally taught for the first time in the program in the Research Methods course. Students did not review the TOSLS questions or their scores until they had completed the practical SL skills assessment twice, and then, they could then reflect on their skill growth throughout the semester (after the online survey had ended).

### Retention of practical SL skills assessment: reassessment of TOSLS after 4 months

At the start of the following academic semester (Fall 2023), 4 months after the end of the Winter 2023 semester, students from the Winter 2023 Research Methods course (that used the flipped classroom teaching and learning approach) were invited to participate and complete another practical SL skills assessment using the 28 TOSLS questions to measure practical SL skill retention. Students from the Winter 2023 Research Methods course were sent a private survey link (using Qualtrics) to their university email address to permit their access to the TOSLS questions. Students provided their informed consent to participate (*n* = 46) and have their practical SL skill retention results connected to their data collected during the Winter 2023 semester in the flipped classroom. Of the 57 students that participated in the research project during the Winter 2023 semester, *n* = 46 students completed the reassessment of their practical SL skills using TOSLS, reflective of 80.7% participation within the study and 54.1% participation from the entire Research Methods class (*n* = 46 of 85 students in the Winter 2023 course).

### Statistical analyses

Statistical analysis was conducted using graphpad prism (GraphPad Software, Inc., La Jolla, CA, USA). Paired *t*‐tests were used to determine changes in endpoints assessed between the start and end of the semester, respectively. One‐way ANOVA followed by Tukey's range test was used to determine changes in TOSLS scores over time (start of Winter 2023 semester, end of Winter 2023 semester, 4 months later at the start of the Fall 2023 semester). Pearson correlations were used for all correlation analyses. A *P*‐value of 0.05 was set to denote statistically significant differences.

## Results

### Changes in students self‐perceived SL skill competency in a flipped classroom learning environment

**Fig. 2:**
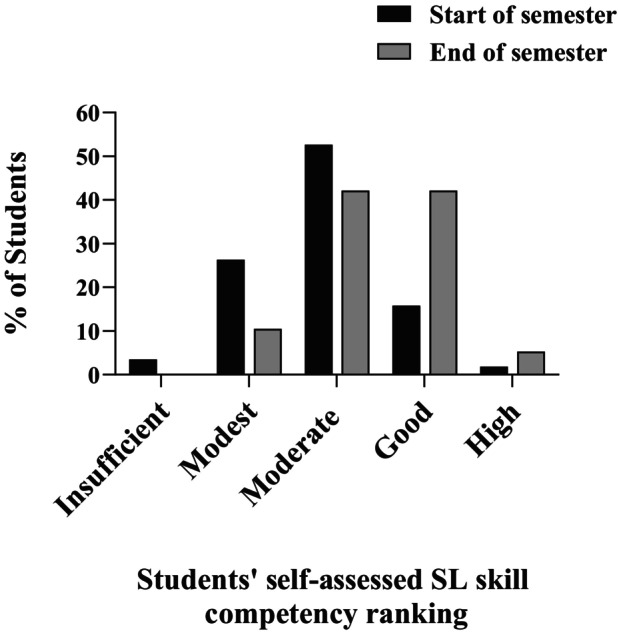
Distribution of students' self‐assessed scientific literacy skill competency (ranging from insufficient to high) at the start (black bars) and end (gray bars) of the semester.

In a single question on the surveys, students' were asked to self‐assess their overall SL skill competency (ranging from insufficient to high) at both the start and end of the semester, as shown in Fig. 2. Initially, 82.4% of students assessed their SL skill competency as moderate or lower and only 17.6% of students assessed their skills as good or high. Conversely, at the end of the semester 47.4% of students assessed their SL skills as good or high, and 52.6% of students assessed their SL skills as moderate or modest, with no students self‐identifying their SL skills as insufficient.

Changes in students' self‐perceived SL skill capabilities for individual components of SL were assessed at the start and end of the semester in the flipped classroom learning environment are shown in Table 1. Students demonstrated increases in their self‐perceived SL capabilities in most SL skill categories, with the most significant gains in their ability to think critically about study designs, assess the design validity, and identify strengths and weaknesses (*P* < 0.05). Furthermore, students also gained confidence in their ability to draw their own conclusions about research study findings and engage with reading the entire scientific paper instead of relying on the content presented in the abstract (*P* < 0.05). The one skill that students did not report increased confidence in their capabilities during the semester was the interpretation of results presented in graphical form (*P* > 0.05).

**Table 1:** Changes in students' self‐perceived scientific literacy skill competency during the academic semester in a flipped classroom learning environment. Data are presented as means (standard error of the mean).

	Survey #1	Survey #2	Mean change	*P*‐value
I feel confident in my ability to…				
Critically assess the validity of research study designs	6.11 (0.24)	7.59 (0.15)	+1.48	< 0.001*
Identify the strengths of a research study	6.69 (0.21)	8.00 (0.16)	+1.31	< 0.001*
Identify the weaknesses of a research study	6.41 (0.25)	7.98 (0.16)	+1.57	< 0.001*
Think critically about the study design in a scientific paper	6.14 (0.28)	7.40 (0.17)	+1.26	< 0.001*
Think critically about the results in a scientific paper	6.89 (0.18)	7.66 (0.18)	+0.77	< 0.003*
Design a research study to address a knowledge gap in the literature	5.35 (0.30)	7.07 (0.21)	+1.72	< 0.001*
Understand the methods that were used in a scientific paper	7.22 (0.16)	7.95 (0.15)	+0.76	0.003*
Interpret results presented in graphs when reading a scientific paper	6.89 (0.26)	7.23 (0.25)	+0.34	0.108
Interpret results presented in tables when reading a scientific paper	7.20 (0.21)	7.59 (0.22)	+0.39	0.044*
Understand the content of the discussion section when reading a scientific paper	6.87 (0.20)	7.68 (0.19)	+0.81	< 0.001*
When reading a scientific paper…				
I draw my own conclusions about the study findings	6.11 (0.30)	7.14 (0.28)	+1.03	0.008*
I read the entire paper and do not rely only on the information from the abstract	5.78 (0.35)	7.23 (0.26)	+1.45	< 0.001*

* Statistically significant differences (*P* ≤ 0.05) between the mean score at the beginning and end of the Winter 2023 semester. The survey scale was from 1 to 10 for each individual question, wherein 1 represented the lowest level of agreement and 10 represented the highest level of agreement.

### Changes in practical SL skills during the semester in flipped classroom learning environment

Changes in students' practical SL skills assessed using TOSLS [1] during the academic semester in a flipped classroom learning environment are shown in Table 2. Overall, TOSLS scores significantly increased during the semester by 16.3%, and students showed gains in multiple individual practical SL skill categories by the end of the semester. Specifically, these skills included significantly increased capabilities in identifying a scientific argument, the validity and use (or misuse) of scientific information, understanding of research study designs and data interpretation (including graphical representations of data), comprehension and interpretation of statistics, and interpreting and applying quantitative data. Interestingly, despite increased skill competency in students' ability to create graphical representations of data and solve problems using quantitative skills, the outcome did not reach statistical significance and could reflect knowledge gaps where additional practice and instructional emphasis are required.

**Table 2:** Changes in students' practical scientific literacy skills assessed using TOSLS during the flipped classroom academic semester. Data are presented as the percentage of Test of Scientific Literacy Skills (TOSLS) questions answered correctly at the start and end of the academic semester.

	TOSLS score at semester start	TOSLS score at semester end	Mean change in TOSLS scores	*P*‐value
TOSLS total score	60.9%	77.2%	+16.3%	< 0.001*
Individual TOSLS skill categories				
Identify a valid scientific argument (Skill #1)	74.1%	86.2%	+12.1%	0.004*
Evaluate the validity of sources (Skill #2)	52.4%	70.3%	+17.93%	< 0.001*
Evaluate the use and misuse of scientific information (Skill #3)	79.3%	95.4%	+16.1%	< 0.001*
Understand elements of research design and how they impact scientific findings/conclusions (Skill #4)	58.6%	80.6%	+22.0%	< 0.001*
Create graphical representations of data (Skill #5)	44.8%	58.6%	+13.8%	0.059
Read and interpret graphical representations of data (Skill #6)	65.5%	83.2%	+17.7%	< 0.001*
Solve problems using quantitative skills, including probability and statistics (Skill #7)	63.2%	71.8%	+8.6%	0.054
Understand and interpret basic statistics (Skill #8)	50.0%	66.1%	+16.1%	0.009*
Justify inferences, predictions, and conclusions based on quantitative data (Skill #9)	49.1%	69.0%	+19.9%	0.003*

* Statistically significant differences (*P* ≤ 0.05) between the start and end of the academic semester for the TOSLS total score or each individual TOSLS skill.

### Relationships between practical SL skills, final grade in the course, and student visits to the course website

Correlative analyses were conducted to determine the relationships between students' practical SL skills and final grades in the course, which are shown in Table 3. Total TOSLS scores were moderately positively correlated with final grades in the course (*r* = 0.526; *P* < 0.001). Analysis of individual TOSLS skill questions answered correctly, and final grades in the course revealed low to moderate positive relationships for all skill categories and final grades (*P* < 0.05). The one practical SL skill that showed no relationship with the final grade in the course was the ability to evaluate the use and misuse of scientific information (*P* = 0.340).

**Table 3:** Correlations between practical scientific literacy skills (i.e., TOSLS scores) at the end of the flipped classroom semester and final grade in the course. Correlative analyses were conducted between students' overall practical scientific literacy skills, namely total Test of Scientific Literacy Skills (TOSLS) scores and individual TOSLS skill category scores at the end of the semester with their final grade in the course. Pearson correlation coefficients (*r*) and *P*‐values are shown.

	Final grade	
	*r*	*P*
TOSLS total score	0.526	< 0.001*
Individual TOSLS skills		
Identify a valid scientific argument (Skill #1)	0.297	0.024*
Evaluate the validity of sources (Skill #2)	0.407	0.002*
Evaluate the use and misuse of scientific information (Skill #3)	0.128	0.340
Understand elements of research design and how they impact scientific findings/conclusions (Skill #4)	0.440	0.009*
Create graphical representations of data (Skill #5)	0.314	0.017*
Read and interpret graphical representations of data (Skill #6)	0.482	< 0.001*
Solve problems using quantitative skills, including probability and statistics (Skill #7)	0.523	< 0.001*
Understand and interpret basic statistics (Skill #8)	0.310	0.018*
Justify inferences, predictions, and conclusions based on quantitative data (Skill #9)	0.344	0.008*

* Statistically significant relationships (*P* ≤ 0.05).

Frequency of accessing the course website to engage with course materials/resources was measured, which could reflect students' engagement in the course. The only data tracked within the course learning management system is the number of times that students accessed the course website during the 12‐week semester. The average number of course website visits was 53.0 ± 1.3, indicating that on average students were accessing the course website more than four times per week during the semester. Correlative analyses showed that the frequency of accessing the course website had no relationship with students' practical SL skills at the end of the semester (*r* = 0.035; *P* = 0.177) or final grade in the course (*r* = 0.011; *P* = 0.445).

### Changes in students' learning approach during the flipped classroom learning environment and the relationship with practical SL skills and final grade in the course

**Fig. 3:**
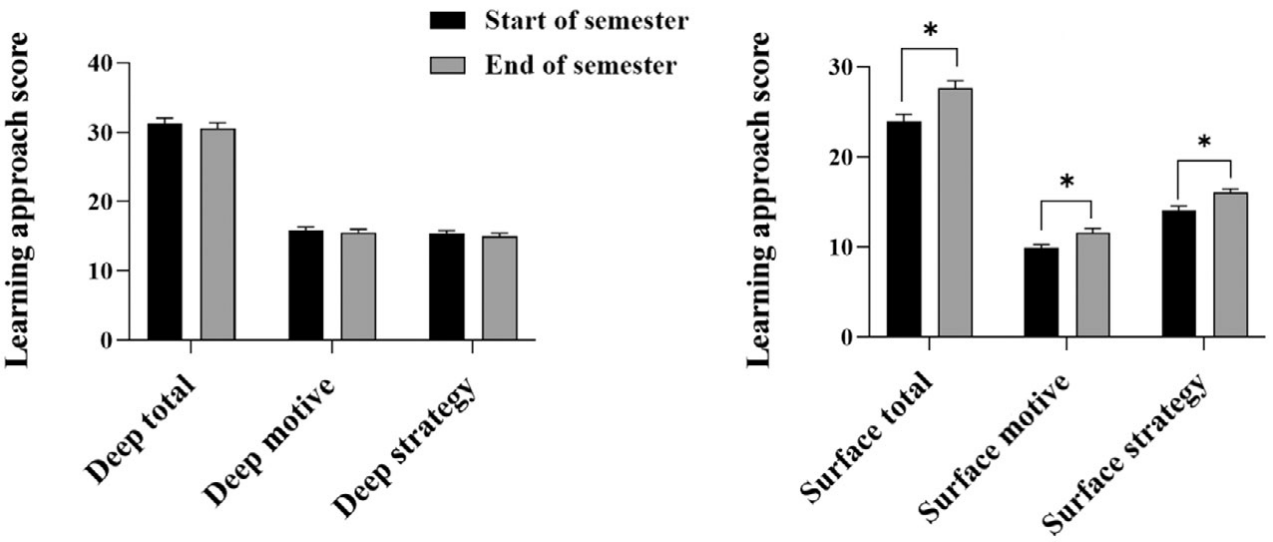
Changes in deep and surface learning approach total, motive, and strategy scores between the start (Survey #1, black bars) and the end (Survey #2, gray bars) of the flipped classroom semester. Bars represent mean values ± SEM. Data were analyzed by a paired *t*‐test and bars marked with an asterisk (*) denote statistically significant differences between the start and end of the semester (*P* ≤ 0.05).

Learning approaches, namely deep and surface learning approaches during the semester, are shown in Fig. 3. Deep learning approach scores did not change (*P* > 0.05). Conversely, surface learning approach scores unexpectedly increased during the academic semester in the flipped classroom learning environment (*P* < 0.05). There were no significant relationships between students' surface learning approach total score and either their overall TOSLS score or their score within each individual TOSLS skill category (*P* > 0.05; Table 4). Conversely, students' deep learning approach total scores were positively correlated with two specific SL skills, namely the ability to evaluate the validity of sources, and the ability to create graphical representations of data (*P* < 0.05; Table 4). Therefore, students who utilized a deep learning approach had greater practical skill development in these two practical SL skill categories that were emphasized in the active learning portion of the flipped classroom learning environment. Students' final grade in the course was positively correlated with deep learning approach total scores (*P* < 0.05; Table 4), whereas there was no significant relationship between final grades and surface learning approach total scores. Collectively, these data emphasize the importance of a deep learning approach, which was associated with greater development of SL skills in specific skill categories and earning higher final grades in the course.

**Table 4:** Correlations between surface and deep learning approach total scores at the end of the flipped classroom semester with practical scientific literacy skills (TOSLS overall and individual skill category scores) and final grade in the course. Correlative analyses were conducted between students' surface and deep total learning approach scores and their practical scientific literacy skills (i.e., Test of Scientific Literacy Skills (TOSLS) overall score and individual TOSLS skill category scores) at the end of the flipped classroom semester and final grade in the course. Pearson correlation coefficients (*r*) and *P*‐values are shown.

	Surface learning approach total score		Deep learning approach total score	
	*r*	*P*	*r*	*P*
Practical SL skills				
TOSLS overall score	0.157	0.160	0.199	0.073
Individual TOSLS skill categories				
Identify a valid scientific argument (Skill #1)	−0.040	0.723	−0.036	0.747
Evaluate the validity of sources (Skill #2)	0.199	0.073	0.225	0.043*
Evaluate the use and misuse of scientific information (Skill #3)	0.100	0.373	0.116	0.300
Understand elements of research design and how they impact scientific findings/conclusions (Skill #4)	0.082	0.465	0.061	0.584
Create graphical representations of data (Skill #5)	0.134	0.229	0.235	0.034*
Read and interpret graphical representations of data (Skill #6)	0.124	0.266	0.173	0.121
Solve problems using quantitative skills, including probability and statistics (Skill #7)	0.019	0.868	0.035	0.755
Understand and interpret basic statistics (Skill #8)	0.078	0.486	0.141	0.208
Justify inferences, predictions, and conclusions based on quantitative data (Skill #9)	0.194	0.081	0.207	0.063
Final grade	0.099	0.376	0.268	0.015*

* Statistically significant relationships (*P* ≤ 0.05).

### Retention of practical SL skills

**Fig. 4:**
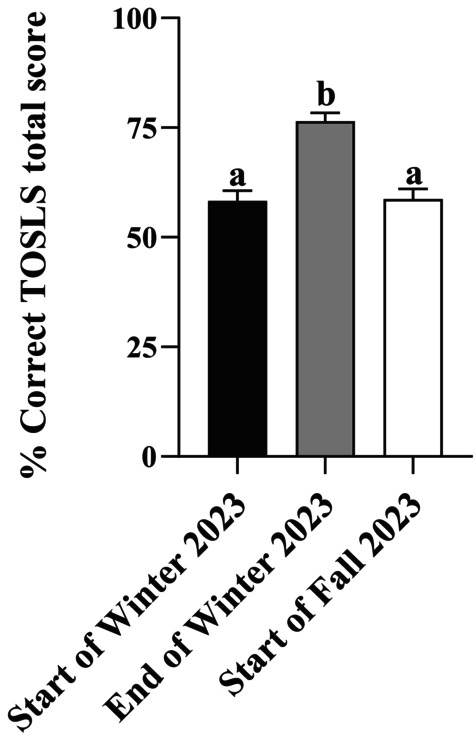
Changes in practical scientific literacy skills (i.e., % of questions correctly answered Test of Scientific Literacy Skills (TOSLS) Score) over time between the start of the Winter 2023 semester (black bars), end of the Winter 2023 semester (gray bars), and 4 months later at the start of the Fall 2023 semester (white bars). Bars represent mean values ± SEM. Data were analyzed by one‐way ANOVA followed by Tukey's range test and bars not sharing a lowercase letter (a or b) are different (*P* ≤ 0.05).

A reassessment of students' practical SL skills using TOSLS was conducted 4 months later at the start of the Fall 2023 semester, as shown in Fig. 4. Upon reassessment, students' overall TOSLS scores decreased compared to the end of the Winter 2023 semester (i.e., the end of the flipped classroom semester) and did not differ from the baseline TOSLS scores from the start of the Winter 2023 semester (*P* > 0.05). This was apparent for all 9 of the TOSLS skill categories assessed, as shown in Fig. S1.

**Fig. 5:**
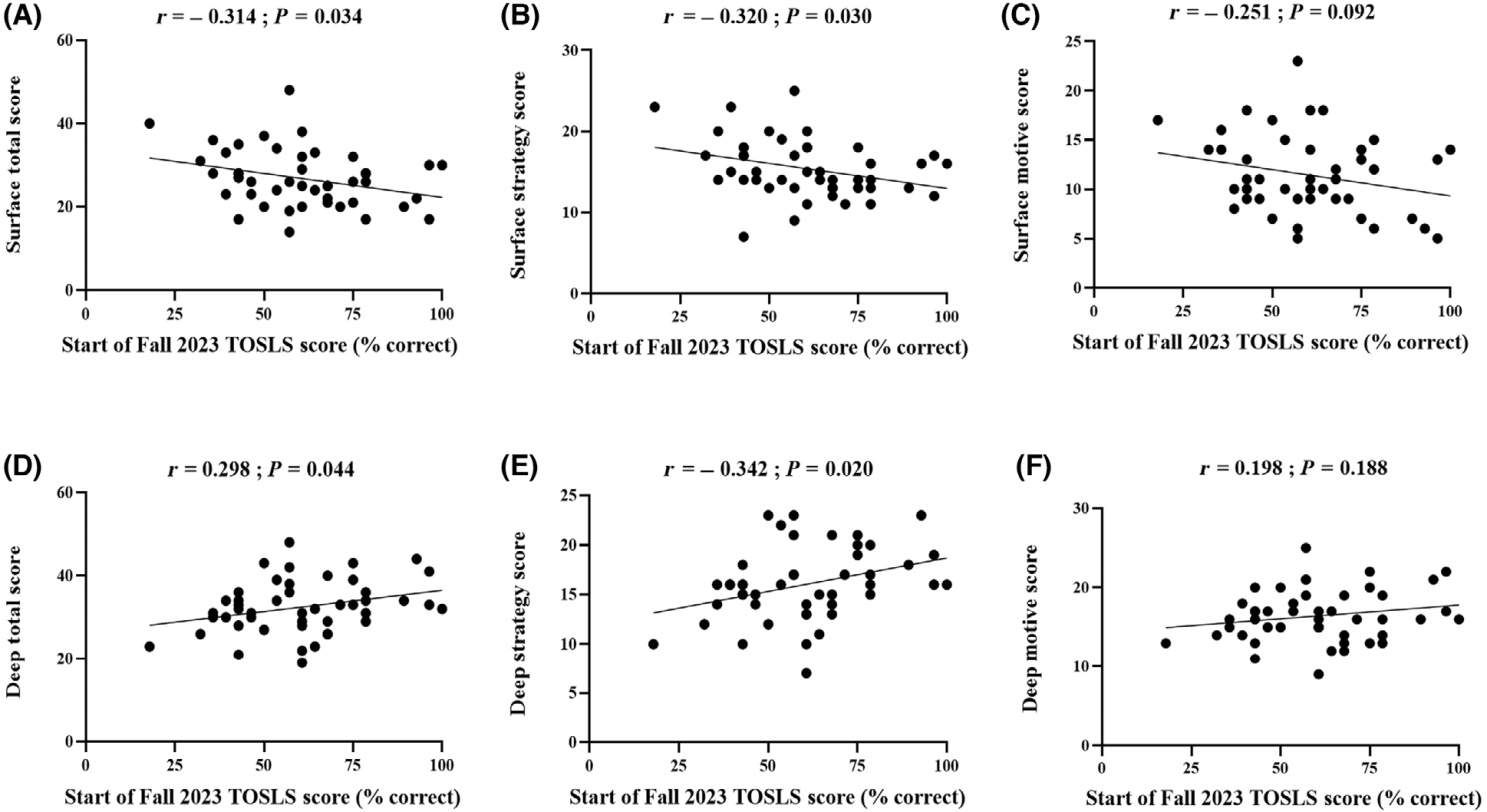
Scatterplots depicting correlative relationships between students' overall Test of Scientific Literacy Skills (TOSLS) scores reassessed at the start of the Fall 2023 semester (i.e., 4 months after the flipped classroom learning environment) and learning approach scores. (A) Surface learning total score, (B) surface strategy score, (C) surface motive score, (D) deep learning total score, (E) deep strategy score, and (F) deep motive scores. Pearson correlation coefficients (*r*) and *P*‐values are shown.

Correlative analyses showed a negative relationship between students' overall TOSLS scores at the start of the Fall 2023 semester and either their surface learning total scores or surface strategy scores (*P* < 0.05; Fig. 5). Conversely, reassessed overall TOSLS scores were positively correlated with both deep learning total scores and deep strategy scores (*P* < 0.05; Fig. 5). Collectively, this indicates that students who implemented a deep learning approach within the flipped classroom learning environment retained more SL practical skill competencies, whereas students utilizing a surface learning approach had lower retention of practical SL skills.

## Discussion

The flipped classroom instructional model encourages students to utilize interactive learning approaches through peer‐led discussions, with an emphasis on developing problem‐solving skills that can address knowledge gaps in formal kinesiology training [43]. In the flipped classroom learning environment associated with the current study, peer‐led discussions centered around kinesiology simulations, case studies, and group discussion questions. The purpose of this study was to determine if engaging in the flipped classroom learning environment influenced students' (a) perceived and/or practical SL capabilities and (b) learning approach, namely promoted deeper learning approaches centered on comprehension and integration of concepts instead of reliance upon memorization and surface learning approaches. As a secondary outcome, practical SL skills were reassessed 4 months later to determine if the skills developed within the flipped classroom learning environment, wherein SL and research methods are first formally taught in the kinesiology program, were retained after the summer semester break (i.e., 4 months later).

Scientific literacy skills are vital components of undergraduate science education and are required in real‐world clinical and professional settings outside of the classroom [1]. Students' self‐perceptions of their SL skill capabilities increased during the semester in a flipped classroom learning environment (Table 1). Self‐assessment of students' skills has been shown to be a valuable educational activity that can lead to improved academic performance, motivation, and critical thinking skills, with both students and faculty perceiving self‐assessment to be a useful approach for promoting developing self‐directed learning skills [44, 45]. Importantly, practical SL skills increased substantially between the start and end of the semester in the flipped classroom learning environment, including an overall 16.3% increase in TOSLS scores and increases in individual SL skills (i.e., TOSLS skill categories) that ranged from 12.1% to 22.0% (Table 2). Interestingly, during the semester students' perceptions of their SL skills did not show a significant improvement in one specific skill, the ability to interpret results presented in graphical form (Table 1), however, this was not in alignment with their practical SL skill capabilities for graphical data interpretation, which increased by 17.7% (Table 2). It is common for students in STEM to experience challenges with data interpretation from visual representations, not only in graphical form but also in charts and diagrams [46], therefore, it is not unexpected that student's perceptions of their graphical data interpretation skills did not align with the gains they showed in practical skill competency. Misalignment of students perceived capabilities and practical skills is common, particularly with underestimates of skill competencies that are a reflection of students' limited confidence in their abilities in contrast with over‐estimators that are more optimistic but can be less critical or realistic in their evaluation [47, 48]. Previously, the flipped classroom approach has been shown to increase students' motivation to learn, and consequently, they may be more invested in the course content during the knowledge acquisition phase [49]. This may serve as a potential explanation for students being naturally more engaged with course‐related tasks and exhibiting better comprehension of course concepts. Previous studies have demonstrated how the flipped classroom learning environment can promote critical thinking, problem‐solving, creativity, communication, collaboration, and information literacy skill development, which can help students transition from the classroom to the professional workforce [18, 33]. The findings from the current study add practical SL skill development to the list of workplace‐relevant skills developed within the flipped classroom learning environment.

Students' TOSLS total score at the end of the semester was positively correlated with their final grade in the course (Table 3), indicating that students that had developed greater practical SL capabilities had higher academic achievement in the course. Therefore, in the flipped classroom learning environment that centered on practicing and utilizing SL skills, the students that engaged in these activities and experienced greater gains in their SL practical skills utilized these acquired skills to achieve higher grades in the course. With respect to specific SL skill categories, positive relationships were also observed between students' practical SL skill capabilities in all of the TOSLS skill categories and their final grade in the course, except for their ability to evaluate the use and misuse of scientific literature (Table 3). This highlights an area for further skill development that is highly relevant in higher education in science disciplines given that SL skills are necessary to combat the spread of misinformation through critical analysis of scientific information to make well‐informed decisions and assess the credibility of scientific data [50, 51, 52, 53].

Assessment of students' retention of critical skills and knowledge after a learning experience or course is important because at the program level this can inform instructors about knowledge gaps and identify skills or concepts that need to be reintroduced or continually reinforced in subsequent courses and/or program years. In the undergraduate kinesiology program associated with the current study, SL skills are formally taught and practiced for the first time in the Research Methods course, therefore, an assessment of students' retention of practical SL skill competency was an important secondary objective. Four months after the flipped classroom learning experience (which was the summer semester when students do not take any courses), the re‐evaluation of practical SL skills using TOSLS was shown to revert to baseline (i.e., the start of the winter semester and the flipped classroom learning experience in the Research Methods course; Fig. 4). Although it may appear that SL skills gained through the flipped classroom learning environment were lost over the summer semester, it is important to consider that other skills beyond SL would be anticipated to be developed in a flipped classroom learning environment but were not evaluated in the current study. Further, although comprehensive, the TOSLS SL evaluation tool does not capture all elements that are part of the broad spectrum of SL skills [1, 2, 3, 4, 5], and therefore, were not assessed in the current study. The erosion of knowledge and skill retention in university students has been demonstrated over extended breaks, such as the summer academic semester, and this decrease in skill competency and/or academic performance becomes more pronounced over longer periods of time [54]. The SL retention data highlight the need for reinforcement of SL concepts and continual educational opportunities to reintroduce concepts, practice, and further develop SL skill competencies throughout the undergraduate program. Previously the reassessment of SL practical skills using TOSLS in fourth‐year biological science students showed that skill competency was maintained after 8 months [40], however, these students gain SL skills from multiple courses and years of educational experience where SL skills are introduced and then reinforced. Consequently, the practical SL skills (i.e., TOSLS scores) in fourth‐year students were higher than students' scores in the current study [40], which suggests that SL skills may be less likely to decline with ongoing academic reinforcement. In contrast, students in the current study were in their second year and were formally introduced to SL concepts and given opportunities to practice and develop SL skills for the first time in their undergraduate program in the research methods course in this study, and therefore, students exhibited lower TOSLS scores. It would be expected that students would need to have concepts reintroduced in subsequent educational experiences, and in this connection, practical SL skills are continually reinforced in the subsequent years of the program. Collectively, these results highlight the need for continual skill evaluation to inform academic program curricular quality improvement. Skill development is a gradual and iterative process that will differ for each student, and therefore, individual students' SL skill retention will also differ, despite the loss of skills on an aggregate basis in the course. Importantly, correlative analyses determined that students with higher deep learning approach total scores or deep learning strategy scores had higher practical SL skills upon TOSLS reassessment 4 months after the flipped classroom learning experience (Fig. 5). Conversely, students with higher surface learning approach total scores or surface learning strategy scores had lower practical SL skill retention (Fig. 5). These data highlight the relevance and value of a deep learning approach to promote not only skill development (Table 3) but also critical skill retention (Fig. 5). Instructional approaches to promote deeper learning behaviors (e.g., highly engaged, motivated, interested, seeking a deeper understanding of course content and exhibiting greater self‐regulation in learning) and discourage surface learning approaches (e.g., minimal engagement and learning by rote memorization tactics with reduced retention of information) [42] would be beneficial for promoting skill development in undergraduate education, in both the flipped classroom and traditional lecture learning environments.

Multiple factors influence students' learning approach including student‐derived factors such as learning and studying behaviors, learning engagement, preexisting knowledge of the content, and learning environmental factors that can include instructors' teaching approach and the learning environment itself [42]. Typical traits of students utilizing a deep learning approach include higher engagement with their learning, self‐regulation, interest in course concepts or an assigned task, and the ability to use their time more efficiently [42, 55], which are skills that are required to optimally engage in a flipped classroom learning environment. Conversely, students utilizing a surface learning approach complete the bare minimum requirements of a task and tend to learn via rote memorization [42], which is an approach less conducive for the knowledge application and engagement expectations during class time in a flipped classroom learning environment. Therefore, evaluation of changes in students' learning approach during the experience of a flipped classroom learning environment that promotes engagement with the course concepts, problem‐solving, application of learning and synthesis, and collaborating with peers [28, 56, 57] is warranted. In the current study, deep learning approach total scores, deep motives, and deep strategy scores did not change during the academic semester (Fig. 3), indicating that students utilizing a deep learning approach continued to do so throughout the semester. Conversely, surface learning approach scores increased between the start and end of the semester (assessed in the final week of the semester, week 12), which may reflect a strategy employed by students to manage end the of semester workload and course expectations prior to final examinations. Alternatively, the increase in surface learning approach scores could be representative of a change in learning approach that started earlier in the semester as workload and schedule expectations associated with a flipped classroom were new for these students and deviated from the passive nature of attending weekly lectures in a traditional lecture‐based learning environment. Importantly, the assessments (examination questions and assignments) in the course were scaffolded throughout the semester to increase in complexity and skill development according to Bloom's Revised Taxonomy [38], with more emphasis in the early part of the semester on assessment activities centered on recall, understanding and application of knowledge, and later in the semester more emphasis was placed on higher‐order learning behaviors including analysis, evaluation, and creation of original work. Students may have employed surface learning approaches to adapt to the increasing complexity of course assessments, although this was not directly assessed throughout the semester. Thus, a mid‐semester evaluation of learning approach could provide some insight into students learning approach motivations and adaptations to a flipped classroom learning environment, and therefore, further study is required. Importantly, students that had higher deep learning approach total scores assessed at the end of the semester in a flipped classroom learning environment were positively associated with greater SL skills in the ability to evaluate the validity of sources, and creating graphical representations of data, skills that are commonly challenging for students to develop [46, 58].

The increase in surface learning approach scores at the end of the semester may reflect the time in the semester when there are overlapping deadlines in all courses between final assignment due dates and the need to prepare for final examinations. For context, at this University, classes end on the Friday of week 12 and the 2‐week final examination period starts at the beginning of week 13. The increase in surface learning approach scores may also be related to the additional time requirements associated with a flipped classroom learning environment. Expectations for students in the flipped classroom include the time required to watch video lectures before in‐class learning activities, which has been identified to be burdensome by students, particularly those who prefer traditional lecture‐based learning environments [25, 27]. Furthermore, personal attributes that are developed in and promote success in a flipped classroom learning environment align with a deep learning approach, such as engagement, self‐regulation, higher‐order cognitive skills (e.g., application, analysis, and synthesis of information) align with a deep learning approach [42], and therefore, students that struggled with these elements may decrease their engagement in the course, as reflected in the increases in surface learning approaches to meet minimum expectations in the course. Students that struggled with self‐regulation and engagement may not have as beneficial of a learning experience compared to students that utilize a deep learning approach and fully engaged in all elements of the flipped classroom learning experience. This is particularly relevant for students that may have focused their efforts onto one element of the flipped learning environment such as learning/studying emphasis on the lecture videos and placing a lower priority of importance on the in‐class application activities, as seen previously [27]. Adoption of approaches to promote engagement throughout the course and provide support for students experiencing challenges with self‐regulation, schedule organization, motivation, procrastination, and/or introducing elements into the course that increase student accountability for viewing the pre‐recorded lectures and completing any assignments to prepare them for the in‐class application activities are needed. Therefore, student buy‐in with respect to participation and engagement in the flipped classroom learning environment is needed to ensure this teaching approach is effective, and in this connection, collecting student experience feedback for ongoing quality improvement of the flipped classroom learning experience is required, although not evaluated in the current study.

A limitation of this study is the lack of a control group wherein SL skill development during the semester was measured in a traditional lecture format, and the gains in practical SL skills developed during the academic semester could be compared to those measured in the flipped classroom learning environment. Future studies should be conducted to determine if similar gains in practical SL skills are achieved in a traditional in‐person lecture‐based learning environment and a flipped classroom learning environments. Although this comparison would be of interest, given the increases in skill competencies observed during the semester in the flipped classroom learning it may not be appropriate to revert to a traditional lecture format and deny students the learning experience from the flipped classroom. The Research Methods course was taught in a flipped classroom learning environment in the first full semester when in‐person learning was permitted at this university since the start of the COVID‐19 pandemic. The purpose of this study was to determine if the flipped classroom learning environment was a viable teaching and learning approach to promote students' SL skill development, and students' familiarity with an online learning environment could facilitate their adaptation to and engagement with the flipped classroom. This was important, as academic engagement during the COVID‐19 pandemic decreased when students were socially distancing and learning remotely in an online learning environment, although some students were shown to prefer online learning and/or social distancing [59, 60, 61, 62, 63]. During the pandemic, studies showed that students overall academic engagement was adversely impacted by their emotional experience and reduced mental health, including loneliness, anxiety, depression, and/or frustration stemming from the impaired ability to share a physical learning space or uncertainty about pandemic‐associated policies and the future [59, 60, 64, 65, 66]. Therefore, the combined elements of independent online learning/lecture viewing prior to the social interaction in the in‐person active learning component of flipped classroom may have offered a blended learning experience to support students transitioning out of pandemic‐associated remote online learning environment. It is important to note that not all gains in practical SL skills observed during the academic semester can be exclusively attributable to the learning experience in this course and the flipped classroom learning environment, as students were taking other courses simultaneously. However, it should be noted that concepts related to scientific methods, data interpretation, and critical analysis of the scientific literature are formally introduced in this program in the Research Methods course, and therefore, since students take all of the same courses and complete the program as an academic cohort, students in this study would have had similar concurrent learning experiences and previous undergraduate level learning experiences, which may reduce the impact of this potential study limitation. Another limitation is in the assessment of practical SL skill retention, which was only measured after 4 months following the gap in educational experience for the summer semester. Retention of any knowledge or skills has been shown to decrease following a gap in educational experience [54], and retention of skills may have been higher if students were still actively engaged in coursework between SL skill assessments. Importantly, the practical SL skills being evaluated 4 months later are first introduced to students during the Research Methods course, and therefore, these were new concepts for most students. Skill retention levels may have been higher if SL skills were not newly introduced (as in the current study), but instead were skills that had been reinforced through successive educational experiences. Importantly, in this study only practical SL skill competencies were assessed both during the semester and again 4 months later in a skill retention reassessment, however, other critical skills and competencies were not formally assessed in this study which may have also been improved during the flipped classroom semester and may have also been retained during the 4‐month follow‐up period. Therefore, the results pertaining to SL skill development in a flipped classroom learning environment should not be extrapolated to other skill competencies, and further investigation is required.

Collectively, the findings of this study suggest the importance of utilizing the flipped classroom learning environment to facilitate SL skill development. Moving forward, it would be beneficial to accommodate learner preferences and address time constraint limitations to ensure pre‐class material is manageable for students to complete ahead of time. The implementation of this curriculum takes valuable time and effort, however, could ultimately improve the quality of education afforded to students.

## Conflict of interest

The authors declare no conflict of interest.

## Author contributions

JMM and LAS conceived and designed the project. EBKB, AA, SR, CM, LAS, and JMM acquired, analyzed, and interpreted the data. EBKB, SR, and AA prepared the figures and tables. EBKB, SR, LAS, and JMM wrote the paper. All authors edited and approved the final paper.

## Supporting information


**Fig. S1.** Changes in practical scientific literacy (SL) skills (i.e., % of Test of Scientific Literacy Skills (TOSLS) questions answered correctly for each of the 9 TOSLS skill categories) over time.
