# Supplementary material for: Using a flipped classroom teaching and learning approach to promote scientific literacy skill development and retention
Source: FEBS Open Bio. 2024 Dec 3;15(3):490–505. doi: 10.1002/2211-5463.13938 (PMC11891771; doi:10.1002/2211-5463.13938)
Supplement: Supplementary file 1 — Fig. S1. Changes in practical scientific literacy (SL) skills (i.e., % of Test of Scientific Literacy Skills (TOSLS) questions answered correctly for each of the 9 TOSLS skill categories) over time. [file FEB4-15-490-s001.pdf]

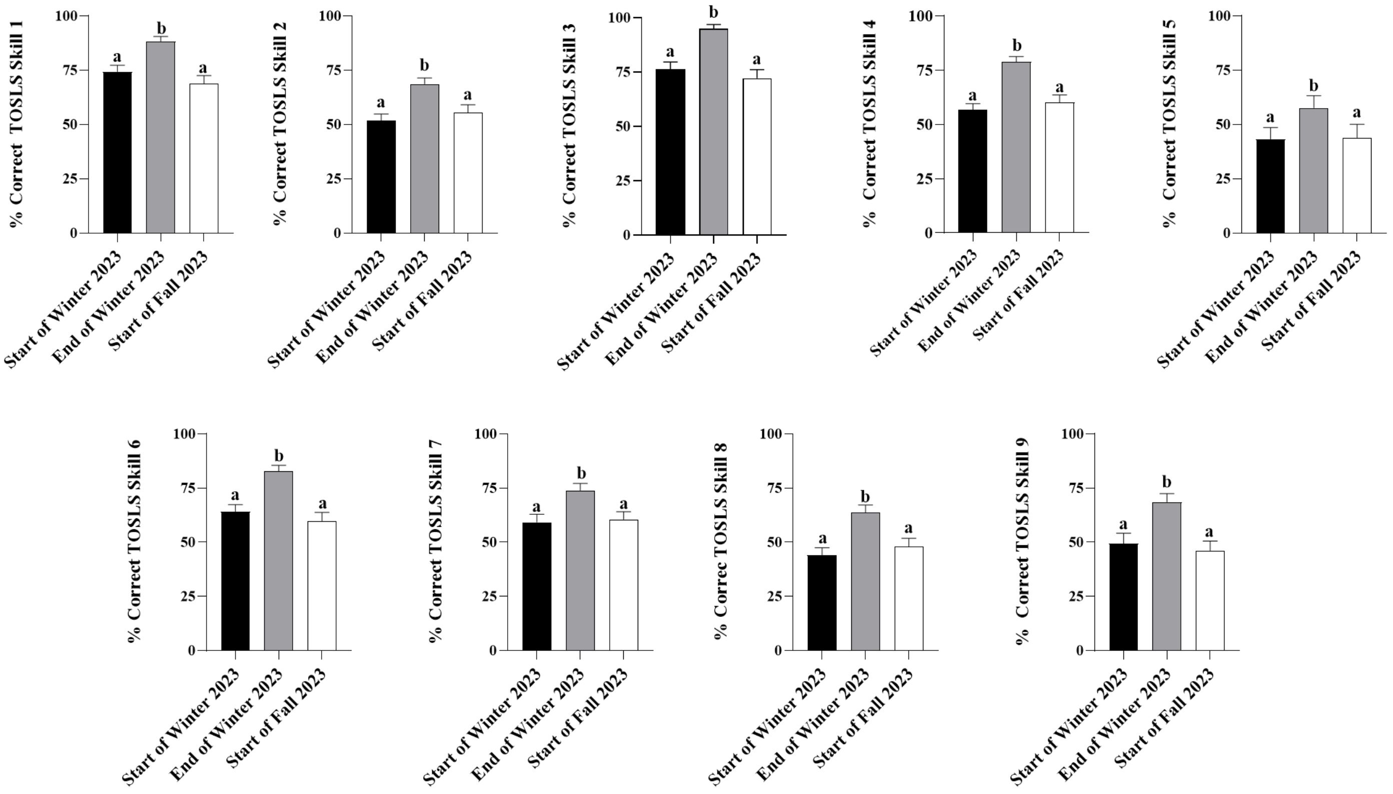

Supplementary Figure 1. Changes in practical scientific literacy (SL) skills (i.e., % of Test of Scientific Literacy Skills (TOSLS) questions answered correctly for each of the 9 TOSLS skill categories) over time. Start of the Winter 2023 semester (black bars), end of the Winter 2023 semester (grey bars) and four months later at the start of the Fall 2023 semester (white bars). Bars represent mean values ± SEM. Data were analyzed by one-way ANOVA followed by Tukey's range test and bars not sharing a lower-case letter are different ($P \leq 0.05$).
